# Complete Genome of *Lactobacillus iners* KY Using Flongle Provides Insight Into the Genetic Background of Optimal Adaption to Vaginal Econiche

**DOI:** 10.3389/fmicb.2020.01048

**Published:** 2020-05-26

**Authors:** Woori Kwak, Young-Hyun Han, Donghyeok Seol, Hyaekang Kim, Hyeonju Ahn, Misun Jeong, Jaeku Kang, Heebal Kim, Tae Hyun Kim

**Affiliations:** ^1^C&K Genomics, Songpa-gu, South Korea; ^2^Priority Research Center, Myunggok Medical Research Institute, College of Medicine, Konyang University, Daejeon, South Korea; ^3^Department of Agricultural Biotechnology and Research Institute of Agriculture and Life Sciences, Seoul National University, Seoul, South Korea; ^4^Department of Pharmacology, College of Medicine, Konyang University, Daejeon, South Korea; ^5^Research Institute of Agriculture and Life Sciences, Seoul National University, Seoul, South Korea; ^6^Myunggok Medical Research Institute, College of Medicine, Konyang University, Daejeon, South Korea; ^7^Department of Obstetrics and Gynecology, Konyang University Hospital, Daejeon, South Korea

**Keywords:** vaginal microbe, *Lactobacillus iners*, long-read assembly, Oxford nanopore, genomic adaptation

## Abstract

Despite the importance of *Lactobacillus iners* and its unique characteristics for the study of vaginal adaption, its genome and genomic researches for identifying molecular backgrounds of these specific phenotypes are still limited. In this study, the first complete genome of *L. iners* was constructed using a cost-effective long-read sequencing platform, Flongle from Oxford Nanopore, and comparative genome analysis was conducted using a total of 1,046 strain genomes from 10 vaginal *Lactobacillus* species. Single-molecule sequencing using Flongle effectively resolved the limitation of the 2nd generation sequencing technologies in dealing with genomic regions of high GC contents, and comparative genome analysis identified three potential core genes (INY, ZnuA, and *hsdR*) of *L. iners* which was related to its specific adaption to the vaginal environment. In addition, we performed comparative prophage analysis for 1,046 strain genomes to further identify the species specificity. The number of prophages in *L. iners* genomes was significantly smaller than other vaginal *Lactobacillus* species, and one of the specific genes (*hsdR*) was suggested as the means for defense against bacteriophage. The first complete genome of *L. iners* and the three specific genes identified in this study will provide useful resources to further expand our knowledge of *L. iners* and its specific adaption to the vaginal econiche.

## Introduction

It is well known that the commensal microbiome in the vaginal tract is closely related to vaginal health, and the healthy vaginal econiche is dominated by a limited number of *Lactobacillus* species in most women (Ravel et al., 2011; Spear et al., 2011). Previous studies identified that the vaginal microbial communities of healthy women were typically dominated by one or few species of lactobacilli (*Lactobacillus crispatus, Lactobacillus iners, Lactobacillus gasseri*, or *Lactobacillus jensenii*) (Hummelen et al., 2010; Srinivasan et al., 2010), and they could promote a healthy vaginal econiche by actively preventing growth and colonization of bacterial, fungal, and viral pathogens (Petrova et al., 2015). Among these *Lactobacillus* species, *L. iners* has been considered as the most prevalent *Lactobacillus* species (Hummelen et al., 2010; Ravel et al., 2011) and known to have very unique characteristics compared to other commensal *Lactobacillus* species in the vaginal econiche.

This organism is known to have rod-shaped cell morphology with Gram-positive characteristics, but unlike other *Lactobacillus* species, it is not always clearly stained as Gram-positive, and some isolates showed coccobacillary morphology rather than bacillary (De Backer et al., 2007; Lebeer et al., 2008). It is unable to grow on de Man-Rogosa-Sharpe agar, a selective culture medium for lactobacilli growth, and nutrient requirement of *L. iners* is known to be more complex than the other vaginal lactobacilli (Rampersaud et al., 2011). Along with its unique phenotypic traits, the genome of *L. iners* is also unusual compared to other *Lactobacillus* species. *L. iners* has the smallest single circular genome (1.3 Mb) among known lactobacilli determined so far, and its size is within the range of the genome sizes of human symbionts and parasites. This small genome size is considered as a result of large scale gene loss and genome reduction during the rapid evolution for specific adaption to the vaginal econiche (Macklaim et al., 2011). A previous genome study of *L. iners* identified that the genome reduction of *L. iners* included the loss of genes involved in the carbohydrate transport and energy metabolism which might be related to its complex nutrient requirements (Macklaim et al., 2011). As opposed to massive gene loss, unlike other lactobacilli, *L. iners* produce an unusual pore-forming cholesterol-dependent cytolysin (CDC) called Inerolysin (INY) which is typically found in Gram-positive pathogenic bacteria (Rampersaud et al., 2011). Unlike other human-specific CDCs such as vaginolysin (VLY) from *Gardnerella vaginalis* or intermedilysin (ILY), INY can be active in the acidic vaginal environment (PH range of 4.5–6.0), and it has a broad range of target species (Rampersaud et al., 2011). The adhesion ability to epithelial surfaces is also one of the important phenotypic traits of vaginal *Lactobacillus* species because it allows colonization and host interaction and excludes pathogens (Osset et al., 2001). Even though the genome of *L. iners* lacks most of the known adhesion factors of *Lactobacillus* species (Morris et al., 2012), it shows a strong adhesion ability to vaginal epithelial cells (McMillan et al., 2013). In addition, *L. iners* is known as the only vaginal *Lactobacillus* species that continued presence in the vagina with the normal or intermediate condition as well as with bacterial vaginosis (BV) as diagnosed by Nugent scores (Burton and Reid, 2002; Verhelst et al., 2005; Tamrakar et al., 2007), and it indicates that *L. iners* seems to be better adapted than other lactobacilli to dynamically changing vaginal environments.

However, in spite of these unique characteristics and small genome size of *L. iners*, high-quality genomes and genomic researches for identifying molecular backgrounds of these specific phenotypes are still limited. The reason why *L. iners* genome still has not been completely sequenced might stem from the fact that the GC contents of this species was significantly lower than the other lactobacilli, including other vaginal lactobacilli (Macklaim et al., 2011; Mendes-Soares et al., 2014). In this study, therefore, we conducted the first complete genome assembly of the *L. iners* species isolated from the healthy vagina of South Korean woman using a latest cost-effective long-read sequencing platform, Flongle from Oxford Nanopore technologies and performed large scale comparative genome analysis with 9 *Lactobacillus* species (*L. crispatus, L. gasseri, L. helveticus, L. jensenii, L, johnsonii, L. plantarum, L, reuteri, L. salivarius, L. vaginalis*) which are previously reported to inhibit the human vagina. We identified potential candidate genes that could be closely related to the unique phenotypes of *L. iners* for its specific adaption in the vaginal econiche, and the complete genome constructed in this study can provide useful resources for future studies.

## Materials and Methods

### Sample Preparation

To isolate *L. iners*, healthy vaginal flora was collected from visiting patients of the outpatient clinic of obstetrics and gynecology of Konyang University hospital. Diluted vaginal flora was cultured on Tryptic Soy Agar plates with 5% defibrinated sheep blood anaerobically using the BD BBL GasPack system (NY, United States) in 37°C during 48 h. Using Tryptic Soy Broth with 5% defibrinated sheep blood, single colonies were grown to get enough amount of DNA for sequencing. According to cell morphology and Gram-staining of isolates, candidate *L. iners* strain KY was selected, and it was confirmed using 16S rRNA sequencing using ABI 3730xl. 16S rRNA sequencing was conducted using 24F-AGAGTTTGATCMTGGCTCAG, 1492R-TACGGYTACCTTGTTACGACTT primers, and the generated forward and reverse reads were merged. Merged full-length 16S rRNA sequence was matched to reference RNA sequence database of NCBI (refseq_rna) (Yatsunyk et al., 2008) using BLASTn for species identification.

### Genome Sequencing and Assembly

DNA was extracted from the cultured bacteria cells using Kit PureHelix Genomic DNA Prep Kit (Solution Type)-Bacteria with minor modification. Briefly, cell pellets were resuspended in 600 μl Cell Resuspension solution with 4 μl Lysozyme (100 mg/ml) and incubated at 37°C for 1 h for Gram-positive bacteria. The cell was re-collected by centrifuging at 12000 rpm for 2 min. The pellet was resuspended in 600 μl Cell Lysis solution containing 3 μl RNase A (4 mg/ml) by pipetting and then lysed at 37°C for 30 min. To remove protein, protein precipitation solution was added to lyse the sample. The clear supernatant after centrifuging was transferred into a new tube and DNA was precipitated by adding 0.8 Vol of Isopropanol. Isolated gDNA was quantified and qualified by gel electrophoresis, 260/280 nm absorbance ratio and Quant-iT PicoGreen dsDNA Assay Kit (Invitrogen).

The library was prepared using the ONT 1D ligation Sequencing kit (SQK-LSK109) with the native barcoding expansion kit (EXP-NBD104) following the manufacturer’s protocol. In brief, genomic DNA was fragmented to target length using g-Tube (Covaris). Fragmented DNA was repaired using the NEBNext FFPE DNA Repair Mix and NEBNext Ultra II End Repair/dA-Tailing Module. The end-prepped DNA was individually barcoded with ONT native barcode by NEB Blunt/TA Ligase Master Mix. Barcoded DNA samples were pooled in equal molar amounts. It was ligated with the adapter using the NEBNext Quick Ligation Module. After every enzyme reaction, the DNA samples were purified using AMPure XP beads (Beckman Coulter). The final library was loaded onto Flongle flow cell (FLO-FLG001, R9.4.1), and sequencing was performed on a MinION MK1b and MinKNOW software (19.06.8).

Base-calling and de-multiplexing for generated fast5 from Flongle were conducted using Guppy (v3.4.3) from Oxford Nanopore technologies. To remove the sequencing artifact and chimeric read, Porechop (v0.2.4)^1^ was employed. Genome assembly was conducted using CANU (v1.8) (Koren et al., 2017) with genomeSize = 1.3 Mb parameter. To correct the errors in the assembled sequence, Nanopolish (v0.11.1) (Loman et al., 2015) was used repeatedly until no correction is available. In addition, Racon (Vaser et al., 2017), Rebaler^2^ and medaka^3^ were also employed to compare the polishing efficiency. Assembled CANU contig was corrected multiple times based on Racon using Rebaler, and two additional rounds of medaka polishing were conducted for reducing insertion and deletion error which can affect the gene prediction. To finish the circular genome of *L. iners*, Simple_circularise.py script^4^ and Circlator (v1.5.5) (Hunt et al., 2015) were used. After making assembled genome of *L. iners* KY to circular form using Simple_circularise.py script, Circlator was used to confirm the circularization, and the start position of the circularized genome was adjusted to dnaA gene manually. Assembled genome was submitted to NCBI database with accession GCA_010748955.1. To identify unassembled regions in the previous studies, 6 scaffold level genomes in Refseq database were mapped to the constructed genome using Minimap2 (Li, 2018).

### Genome Annotation and Comparative Genome Analysis

Genome annotation was conducted using Prokka (v1.14.5) (Seemann, 2014) with –rfam option to enable search for ncRNA. Genomic map of constructed *L. iners* KY genome was constructed using CGVIEW (Grant and Stothard, 2008). For comparative genome analysis, a total of 1,045 available genomes for 10 Lactobacillus species in NCBI Refseq (Haft et al., 2017) data were used (*L. crispatus*: 111, *L. gasseri*: 43, *L. helveticus*: 57, *L. iners*: 23, *L, jensenii*: 37, *L. johnsonii*: 38, *L. plantarum*: 469, *L. reuteri*: 175, *L. salivarius*: 88, *L. vaginalis*: 4). Four genomes were randomly selected from each species to construct the gene cluster for 10 Lactobacillus species using OrthoMCL (Li et al., 2003), and then the identified gene clusters specifically existed in *L. iners* were confirmed in 1,045 genomes based on the Prokka annotation. Using all annotated proteins from 9 *Lactobacillus* species, uniqueness of *L. iners* specific genes was confirmed based on BLASTp (Subject Coverage > 0.5, Identity > 0.5, evalue < 1-e5).

### Comparative Prophage Analysis

Existing prophages in 1,045 genomes of 10 *Lactobacillus* species and L. *iners* KY were identified using ProphET (Reis-Cunha et al., 2019), a standalone prophage detection program. To test the significance of the numbers of prophages in the genomes of the 10 *Lactobacillus* species, Kruskall–Wallis test was used, and the pairwise Wilcoxon Rank-Sum test was conducted to compare between each species with FDR correction. For identification of detected prophage from *Lactobacillus* genomes, 2,169 phage and prophage sequences were downloaded from NCBI refseq database, and detected prophage sequences were matched using BLASTn (Altschul et al., 1990).

## Results and Discussion

### Constructing the Complete Genome of *L. iners* KY

Table 1 shows the features of generated raw data from Flongle. About 89, 134, and 215 Mb were generated for 6, 10, and 20K insert size library, respectively. Based on the *L. iners*’s estimated genome size of 1.3 Mb, 68.48X, 103.72X, and 165.87X coverage data were retained from each library. We tried to generate the same amount of data for each library during data generation, but the amount of generated data was increased according to the increase of insert size. On the contrary, the median read length was decreased according to the increase of insert size. N50 length was 4,639 bp for 6K library, 6,727 bp for 10K library, and 7,778 bp for 20K library which was shorter than our expectation based on the insert size of each library. After removing the adapter and chimeric reads from raw data using Porechop, genome assembly using CANU was conducted. Table 2 shows the results of genome assemblies using CANU for each library. Among 3 libraries with different insert sizes, only 20K library which showed high coverage, and long N50 length was succeeded to construct the chromosome level assembly. Scaffold level assembly (8 scaffolds and 5 scaffolds, respectively) was resulted from CANU assemblies using 6K, 10K libraries, and the number of scaffolds was decreased in accordance with the coverage increase. After conducting Nanopolish for error correction in the assembled genome, total length was increased to 1,357,225 bp, and GC contents was decreased to 33.40%, and assembly statistics after circularization was 1,337,870 bp with 33.39% GC contents. Prokka annotation identified 1,684 CDSs in the polished genome. However, the number of predicted genes was much higher than previously reported genome assemblies of *L. iners*, and the polished assembly contained many pseudogenes originated from assembly errors. This indicated that assembly polishing using Nanopolish could not effectively resolve the remained errors in the assembly. Therefore, to achieve more improved *L. iners* genome assembly, we employed additional assembly polishing tools such as Racon, Rebaler, and Medaka which can be applicable only for Nanopore reads. After polishing using Racon (Reblaer) and two additional rounds with Medaka, the final assembly was 1,339,101 bp with 33.35% GC contents, and the number of predicted genes was decreased to 1,465 genes (CDS: 1,354, rRNA: 18, misc_RNA: 22, tRNA: 70, tmRNA: 1) with reduced pseudogenes. This result indicates that the quality of genome assembly using only Nanopore reads varies depending on the polishing tools, and Medaka, the polishing tool from its manufacturer showed better performance than any other tools applied in this study.

**TABLE 1:** Generated data information for three libraries constructed in this study.

**Library**	**Raw reads**	**Raw bases**	**Coverage**	**Med read length**	**Med read length (Q > 7)**	**N50**	**N50 (Q > 7)**
6K	30,240	89,025,851	68.48 X	2,401	2,445	4,639	4,641
10K	41,973	134,845,410	103.72 X	1,643	1,667	6,727	6,752
20K	70,743	215,632,258	165.87 X	1,220	1,245	7,778	7,823

**TABLE 2:** Summary statistics for assemblies using three libraries with different insert size, polishing and circularization.

	**6K**	**10K**	**20K**	**Nanopolish**	**Racon rebaler**	**Medaka**	**Circularized**
Number of Contigs	8	5	1	1	1	1	1
Number of A’s	458,905	462,427	450,114	452,166	451,952	452,057	446,526
Number of C’s	227,647	232,184	225,694	226,022	225,501	225,815	223,631
Number of G’s	236,578	235,744	227,014	227,402	226,791	227,124	222,990
Number of T’s	453,444	460,035	449,647	451,665	451,614	451,716	445,954
Sum	1,376,574	1,390,390	1,352,469	1,357,255	1,355,858	1,356,712	1,339,101
GC contents	33.73%	33.66%	33.48%	33.40%	33.36	33.38	33.35
Minimum	7,185	7,593	1,352,469	1,357,255	1,355,858	1,356,712	1,339,101
Maximum	832,263	1,058,344	1,352,469	1,357,255			
Average	172,072	278,078	1,352,469	1,357,255			
N50	832,263	1,058,344	1,352,469	1,357,255			

**FIGURE 1:**
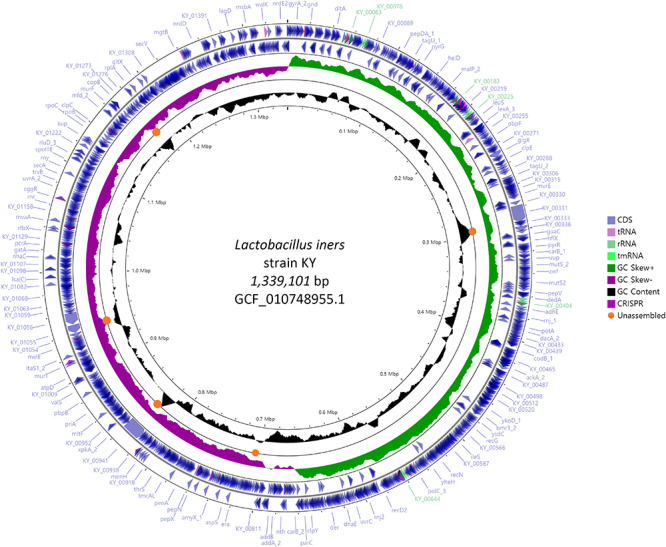
Circular genome map of *L. iners* KY using Prokka and CGVIEW. The arrow direction of CDS shows the location of the gene in the genome. Five colors including bright purple indicate annotated features from Prokka. Orange circles indicate the commonly unassembled region in six previous scaffold level assembly.

We compared the complete genome constructed in this study with the previously reported 6 scaffold level genomes to identify the unassembled region and its characteristics. Orange circles in Figure 1 indicate commonly unassembled regions in previous scaffolds level assemblies. GC contents of those regions were higher or lower than the average. *L. iners* has the smallest genome size among the known *Lactobacillus* species, but complete and chromosome level genomes are hardly available for this small genome. This indicates that GC contents can be one of the reasons for the failure of complete genome assembly in earlier studies. Previous genome assemblies for *L. iners* were conducted using 2nd generation sequencing platforms such as 454 from Roche and Hiseq from Illumina. Since these kinds of platforms use PCR (emulsion PCR for Roche and Bridge PCR for Illumina) for its data generation process, data generation for specific genomic regions with exceptionally high or low GC contents can be difficult. But single-molecule sequencing platforms such as Nanopore and Pacbio which do not employ PCR for its data generation can reduce this limitation, and another recent successful chromosome level assembly of *L. iners* LI335 using Pacbio platform showed the effectiveness of single-molecule sequencing. Especially, Flongle platform from Oxford Nanopore technologies is cheaper than any other sequencing platform currently available in the market, and it can generate about 1 Gb data of almost 200X coverage, based on 5 mb microbial genome. Even though a high error rate is still challenging, the future improvement in accuracy and data throughput of Flongle with proper error correction algorithms and tools can provide more cost-effective and easy ways for constructing complete genomes of various kinds of unknown microbes.

### Comparative Genome Analysis and Specific Genes in *L. iners*

Using OrthoMCL with 40 *Lactobacillus* genomes (4 randomly selected from each 10 vaginal *Lactobacillus* species), a total of 7,453 gene clusters were constructed. We started with 40 genomes because OrthoMCL could not handle whole 1,046 *Lactobacillus* genome used in this study. Among 7,453 gene clusters, 174 clusters were specifically identified in *L. iners* genomes, and most of them encoded hypothetical proteins. And then, further filtering was conducted based on the Prokka annotation results of 1,046 strain genomes to identify unique core genes of all *L. iners* genomes among vaginal *Lactobacillus* species, and three specific genes uniquely existed in all *L. iners* genomes remained. Table 3 shows three specific genes (INY, ZnuA, and *hsdR*) which commonly and uniquely existed in the *L. iners* genomes, and the detailed sequence information for each gene is summarized in Supplementary Table 1.

**TABLE 3:** *Lactobacillus iners* specific genes cluster identified in comparative genome analysis.

**Gene symbol**	**Gene name**	**EC number**
INY	Inerolysin (Pneumolysin)	
ZnuA	High-affinity zinc uptake system binding-protein ZnuA Type I	
*hsdR*	Type I restriction enzyme EcoR124II R protein	EC:3.1.21.3

Inerolysin (INY) is a well known pore-forming toxin that is specifically produced by *L. iners*, and it is included in the cholesterol-dependent cytolysins (CDCs) (Rampersaud et al., 2011; Christie et al., 2018). It binds to the cell membrane and forms oligomeric complexes inserted into the lipid bilayer to make aqueous pores (Flanagan et al., 2009). According to the Black Queen Hypothesis, organisms tend to lose the capacity to synthesize metabolites if they are provided by their hosts or community members, and it can account for gene loss and genome reduction (Morris et al., 2012). During the adaption process to the vaginal econiche, large scale gene loss with horizontal gene acquisition occurred in *L. iners* (Macklaim et al., 2011), and it is highly possible that remaining unique core genes are closely related to the specific adaption of *L. iners* to the vaginal econiche. The vaginal environment is not simple because the fluctuation of hormone can affect mucus and glycogen production, PH, and microbial species which might provide essential nutrients for *L. iners*. The previous study showed that genomes of 11 *L. iners* strains commonly contain this pore-forming toxin gene (Rampersaud et al., 2011), and our study using all available *L. iners* genomes also showed that 24 *L. iners* genomes also commonly had this gene, and this specific type of CDC only existed in *L. iners* among 10 *Lactobacillus* species. This indicates that inerolysin may be one of the essential genes for *L. iners* to stably obtain essential nutrients from host and live in dynamically changing vaginal echoniche. Therefore, it can be used as a potential target gene for specific modulation of *L. iners* and related microbe species in the vaginal flora.

High-affinity zinc uptake system binding-protein ZnuA Type I (ZnuA) is one of the components of ZnuABC, the high-affinity transporter specialized for transporting zinc ions (Patzer and Hantke, 1998; Yatsunyk et al., 2008). ZnuA gene, one of the core genes of *L. iners* identified in this study, is highly conserved between strains of *L. iners.* In the moderate conditions when zinc is abundant, zinc uptake is mediated by the low-affinity permease ZupT, a member of ZIP family transporters (Hantke, 2005). However, in environments with very low zinc availability, zinc import is ensured by ZnuABC, and it is one of the important parts of the systems for metal ion homeostasis in bacteria (Patzer and Hantke, 1998; Yatsunyk et al., 2008). In addition, ZnuA is also known to be closely related to the adhesion ability to the epithelial cells in the host, and it is considered as a virulence factor (Gabbianelli et al., 2011; Li et al., 2015). Li et al. (2015) showed that ZnuA was also significantly important for *Pseudomonas aeruginosa* to adhere to polystyrene plates and HeLa cells, and Gabbianelli et al. (2011) showed that inactivation of ZnuA dramatically decreased the adhering ability of *E. coli* O157:H7 to Caco-2 cells. Therefore, given that *L. iners* the only vaginal *Lactobacillus* species that possesses ZnuA gene, this gene may be one of the essential genes for their adaption to the vaginal econiche and the potential key mediator for strong adhesion to the vaginal epithelial cells, such as previously reported fibronectin (Fn)-binding protein (McMillan et al., 2013).

Type I restriction enzyme R protein (*hsdR*) is one of three components in the type I R/M (restriction and modification) system (Loenen et al., 2013), and this system combines the functions of site-specific methylation and restriction activity in one large multimeric protein. Genes of this system typically form operon, but each component from different operons or single genes can be intermixed in combination. This system can provide protection against invading DNA such as foreign plasmids or the DNA of bacteriophage, and it is known to be one of the phage resistance mechanisms for some specific lactic acid bacteria (Allison and Klaenhammer, 1998). For *Lactobacillus* species, *L. helveticus* was reported to have a plasmid-linked R/M system (Clara et al., 1990), and a recent study showed that the phage resistance strain of *L. helveticus* used Type I R/M system as a defense mechanism for bacteriophage invasion (Zago et al., 2017). Based on the Prokka annotation of 1,046 vaginal *Lactobacillus*, we identified some strains of each species also had *hsdR* gene (*L. crispatus*: 16.2%, *L. gasseri*: 9.3%. *L. helveticus*: 28.1%, *L. jensenii*: 94.6%, *L. johnsonii*: 26.3%, *L. plantarum*: 61.0%, *L. reuteri*: 39.8%, *L. salivarius*: 70.5%, *L. vaginalis*: 75.0%). However, the *hsdR* gene sequence of *L. iners* was very unique compared to that of other vaginal *Lactobacillus* species, which had high sequence similarity with *Staphylococcus* genus. Meanwhile, *hsdR* genes from all *L. iners* genomes had high sequence similarity with *E. coli*. Also, most of the *L. iners hsdR* protein sequences were much shorter (300–600 a.a) than the previously known *hsdR* genes (about 1080 a.a), and they contained c-terminal domain of Type I restriction R subunit. It is well known that the number of vaginal *Lactobacillus* species decreases during the progress of bacterial vaginosis (BV), and it is replaced with anaerobic bacteria such as *Gardnerella* species and genital mycoplasmas (Sobel, 2000). Previous studies strongly suggested that bacteriophage was one of the reasons for sudden *Lactobacillus* decrease during BV (Pavlova et al., 1997; Kiliç et al., 2001), and meta-transcriptome study of *L. iners* showed that CRISPR anti-bacteriophage defense system and restriction-modification system were highly upregulated during BV (Macklaim et al., 2013). Only a small number of *L. iners* genomes are known to have cas proteins (Macklaim et al., 2013), and this indicates that the *L. iners* specific *hsdR* gene is one of the potential core genes providing *L. iners* resistance and viability against bacteriophage infection during BV. Further studies on these genes will provide more understanding about the specific adaption of *L. iners* to the vaginal econiche.

### Prophages in *Lactobacillus* Genomes and Adaption to Vaginal Econiche

**FIGURE 2:**
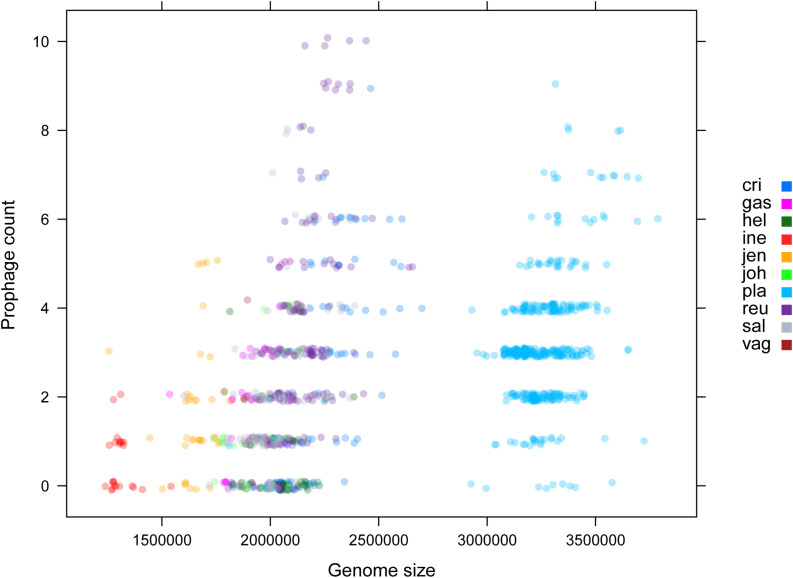
Scatter plot for genome size and identified prophage count using ProphET. Color indicates each vaginal *Lactobacillus* species used in this study.

**FIGURE 3:**
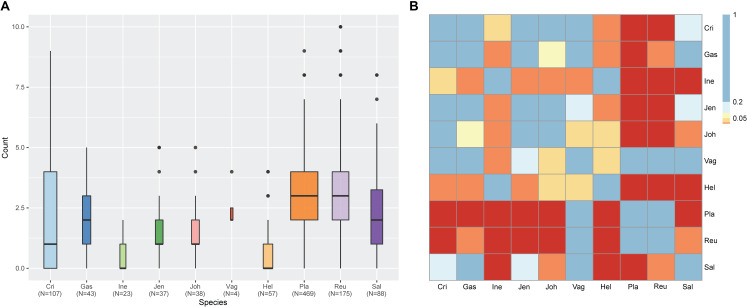
Result of prophage count analysis for 1,046 vaginal *Lactobacillus* species using ProphET. **(A)** Boxplot for the number of prophage in each strain genome. Band width indicate the number of genomes used for plot. **(B)** Heatmap for the result of the pairwise Wilcoxon Rank-Sum test. Colors indicate the significance level with FDR correction. (Blue > 0.2 < Sky blue > 0.1 < Yellow > 0.05 < Orange > 0.01 > Red).

**FIGURE 4:**
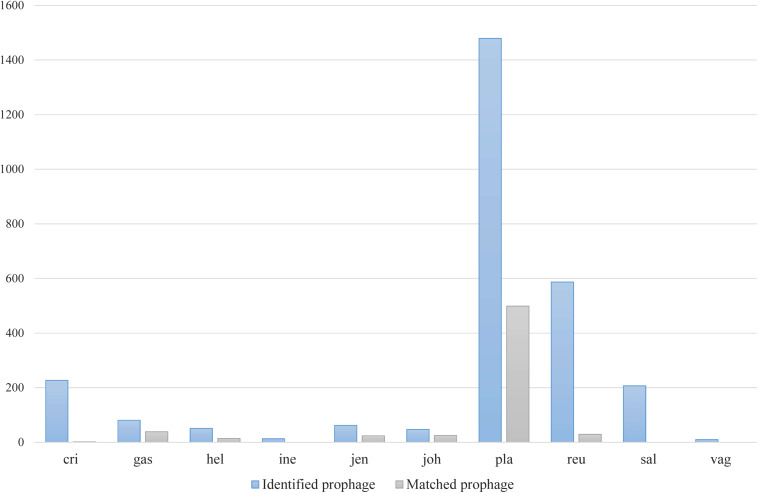
Number of detected and identified prophages using previously known phage and prophage genome sequences.

Among various possible reasons contribute to the decrease of vaginal *Lactobacillus* species during BV, bacteriophage was suggested as one, and Pavlova and Tao (2000) showed that it could be induced from prophage in the *Lactobacillus* genomes. In the comparative gene family analysis using 10 vaginal *Lactobacillus* species, we suggested the *L. iners* specific *hsdR* gene and related Type I R/M system which was upregulated in BV might be one of the key elements for further defense against altered environmental phage load. Therefore, we conducted a prophage analysis for 1,046 genomes of vaginal *Lactobacillus* species to identify the evidence of this hypothesis. Figure 2 shows a scatter plot of the number of identified prophages and the genome size of each species used in this study. There was no significant correlation between the genome size and the number of identified prophages and this indicates that specific defense mechanisms are involved for defense against phage infection. Figure 3A shows a boxplot of the number of prophages that exists in 10 vaginal *Lactobacillus* species. The average numbers of prophage of each 10 vaginal *Lactobacillus* species were different (Kruskall–Wallis test, *p* < 0.05), and genomes of two species (*L. iners* and *L. helveticus*) had significantly smaller numbers of prophage compared to other 8 *Lactobacillus* species (Figure 3B, all pairwise Wilcoxon Rank-Sum test, FDR < 0.05). Two *Lactobacillus* species, *L. iners* and *L. helveticus*, have a significantly smaller number of detected prophages, and they are known to have a type I RM system which can be useful for the defense against bacteriophage invasion. But comparative gene analysis showed that not only these two *Lactobacillus* species but also some strains of other vaginal *Lactobacillus* species used in this study had *hsdR* gene. This result indicates that induced bacteriophage may not be the main reason for the sudden decrease of *Lactobacillus* species during BV. Because even though *L. helveticus* also seemed to have an effective defense system for bacteriophage but it is not the predominant *Lactobacillus* in vaginal econiche and it cannot retain its abundance during BV. Therefore, we can expect more complex reasons for the sudden reduction of vaginal *Lactobacillus* species during the progression of BV and more specific adaption mechanisms in *L. iners* are involved in retaining its abundance during the dynamic environmental change during BV. However, even though bacteriophage may not be the main reason for the sudden reduction of *Lactobacillus* species during BV, it can accelerate the reduction of *Lactobacillus* species and defense systems such as Type I RM system and CRISPR can be useful for viability in changing vaginal environment. Previous transcriptome study observed upregulation of the *hsdR* gene expression in *L.* iners during BV (Miller-Ensminger et al., 2018), and suggested the important role of the *hsdR* gene in some of *L. iners* strains without CRISPR system. Further study will be necessary to identify the role and relatedness of its unique *hsdR* gene for the defense against bacteriophage infection. Figure 4 shows the number of detected and identified prophage in 1,046 genomes. Same as previous study (Miller-Ensminger et al., 2018), most predicted prophage sequences could not be identified its origin even though blast filtering criteria was set to very low (22%: sum of S.Cov > 10%, 5.9%: sum of S.Cov > 50%). Two candidate reasons were expected for this result. Integrated prophage sequences can be weathered during bacterial genome evolution and the current public sequence database does not contain enough sequence information for bacteriophages and prophages. Because bacteriophage is known to have an extraordinary diversity, low similarity (Grose and Casjens, 2014; Pope et al., 2015), 2,169 genomes of bacteriophages and prophages are not enough to fully identify its origin. All identified phages and prophages were matched to *Lactobacillus* phage and prophage, such as *Lactobacillus* phage AQ113, KC5a, Lv-1, phi jlb1, phig1e, Sha1, and *Lactobacillus* prophage Lj928, Lj965, phiadh, etc., and *Lactobacillus* phages are known to have a wide host range for various *Lactobacillus* species (Kiliç et al., 2001). Detected prophages from *L. iners, L. salivarius* and *L. vaginalis* were not matched to previously known phage sequence. All identified prophages in *L. crispatus* (0.88%) and *L. helveticus* (27.45%) were *Lactobacillus* phage AQ113. *Lactobacillus* phage AQ113 was isolated from *L. helveticus* in dairy product and it is included in Myoviridae (Zago et al., 2013). In case of *L. jensenii*, all identified prophages were *Lactobacillus* phage Lv-1 (38.71%), and it is only found in the *L. jensenii* genomes. *Lactobacillus* phage Lv-1 was isolated from vaginal *L. jensenii*, and it is included in Siphoviridae (Martín Rosique et al., 2010). Identified prophages of *L. plantarum* (33.74%) were *Lactobacillus* phage SHA1 (Yoon et al., 2011) and phig1e (Kakikawa et al., 1996), and they are all isolated from *L. plantarum* in previous study. 6 and 4 types of prophages were identified in *L. gasseri* (48.15%) and *L. johnsonii* (38.71%) genomes, and prophages of two *Lactobacillus* species shared its source of origin (Sechaud et al., 1988). Two *Lactobacillus* phage, *Lactobacillus* phage SHA1 and Lj965 from *L. plantarum* and *L. johnsonii* (Ventura et al., 2003), were identified in *L. reuteri* genomes (4.94%). This result suggests that the host range of *Lactobacillus* phage varies according to its type and lineage, and it might be more narrow than previously reported. However, this result has limitations because it was based on the small number of previously reported phage genome sequences, and most of the detected prophage sequences are still unknown. *Lactobacillus* phage is known to be one of the important driving forces of genome evolution (Baugher et al., 2014), and previous study about phageome related to the schizophrenia showed *Lactobacillus* phage is closely related to human health and disease (Yolken et al., 2015). Therefore, accumulation of phage researches and its sequences is necessary for future research and it will help to further expand our knowledge of *L. iners* specific adaption to vaginal econiche and therapeutic modulation using *Lactobacillus* phage.

## Data Availability Statement

The datasets generated for this study can be found in the NCBI, under accession number PRJNA603871.

## Ethics Statement

The studies involving human participants were reviewed and approved by Konyang University Hospital (IRB FILE No: 2017-12-021-010). The patients/participants provided their written informed consent to participate in this study.

## Author Contributions

WK and TK designed the experiments, interpreted the data, drafted the manuscript, and supervised the study. DS, HA, and JK performed the bioinformatic analyses and interpreted the data. Y-HH, HKK, and MJ performed the experiments. HBK contributed to the revision. All authors reviewed the manuscript.

## Conflict of Interest

WK and MJ were employed by C&K Genomics. The remaining authors declare that the research was conducted in the absence of any commercial or financial relationships that could be construed as a potential conflict of interest.

## Footnotes

1 https://github.com/w1bw/Porechop

2 https://github.com/rrwick/Rebaler

3 https://nanoporetech.github.io/medaka/

4 https://github.com/Kzra/Simple-Circularise
