# Supplementary material for: Complete Genome of Lactobacillus iners KY Using Flongle Provides Insight Into the Genetic Background of Optimal Adaption to Vaginal Econiche
Source: Front Microbiol. 2020 May 26;11:1048. doi: 10.3389/fmicb.2020.01048 (PMC7264367; doi:10.3389/fmicb.2020.01048)
Supplement: Supplementary file 1 [file Table_1.DOCX]

Supplementary Table 1. Length, identity and query coverage of 3 specific *L.iners* genes in 23 *L. iners* available genomes used in this study.

| **Accession** | **strain** | **INY** | | | | | |  |
| --- | --- | --- | --- | --- | --- | --- | --- | --- |
|  |  | **nucleotide length** | **Identities** | **Query Coverage** | **amino acids length** | **Identities** | **Query Coverage** | |
| GCF_000149065 | LactinV 11V1-d | 1560 | 99.55 | 100 | 519 | 100 | 100 | |
| GCF_000149085 | LactinV 09V1-c | 1560 | 99.1 | 100 | 519 | 99.61 | 100 | |
| GCF_000149105 | LactinV 03V1-b | 1560 | 99.55 | 100 | 519 | 100 | 100 | |
| GCF_000149125 | LactinV 01V1-a | 1560 | 98.65 | 100 | 519 | 99.23 | 100 | |
| GCF_000149145 | SPIN 2503V10-D | 1560 | 99.29 | 100 | 519 | 99.81 | 100 | |
| GCF_000160875 | DSM 13335 | 1560 | 99.04 | 100 | 519 | 99.81 | 100 | |
| GCF_000177755 | AB-1 | 1560 | 99.04 | 100 | 519 | 99.81 | 100 | |
| GCF_000179935 | LEAF 2053A-b | 1560 | 99.62 | 100 | 519 | 100 | 100 | |
| GCF_000179955 | LEAF 2052A-d | 1560 | 99.49 | 100 | 519 | 100 | 100 | |
| GCF_000179975 | LEAF 2062A-h1 | 1560 | 99.68 | 100 | 519 | 100 | 100 | |
| GCF_000179995 | LEAF 3008A-a | 1560 | 99.55 | 100 | 519 | 99.81 | 100 | |
| GCF_000185405 | ATCC 55195 | 1560 | 99.68 | 100 | 519 | 100 | 100 | |
| GCF_000191685 | UPII 143-D | 1560 | 99.68 | 100 | 519 | 100 | 100 | |
| GCF_000191705 | UPII 60-B | 1560 | 99.36 | 100 | 519 | 99.81 | 100 | |
| GCF_000204435 | SPIN 1401G | 1560 | 99.29 | 100 | 519 | 99.81 | 100 | |
| GCF_000227195 | 7_1_47FAA | 1560 | 98.78 | 100 | 519 | 99.42 | 100 | |
| GCF_001435015 | DSM 13335 | 1560 | 99.04 | 100 | 519 | 99.81 | 100 | |
| GCF_002871595 | UMB0033 | 1560 | 99.17 | 100 | 519 | 99.81 | 100 | |
| GCF_002884695 | UMB1051 | 1560 | 99.36 | 100 | 519 | 99.81 | 100 | |
| GCF_002884705 | UMB0030 | 1560 | 99.62 | 100 | 519 | 100 | 100 | |
| GCF_002892385 | KA00186 | 1560 | 99.62 | 100 | 519 | 100 | 100 | |
| GCF_009556455 | LI335 | 1560 | 99.04 | 100 | 519 | 99.81 | 100 | |
| GCF_902374445 | EMG | 1560 | 98.78 | 100 | 519 | 99.42 | 100 | |

| **Accession** | **strain** | **ZnuA** | | | | | |  |
| --- | --- | --- | --- | --- | --- | --- | --- | --- |
|  |  | **nucleotide length** | **Identities** | **Query Coverage** | **amino acids length** | **Identities** | **Query Coverage** | |
| GCF_000149065 | LactinV 11V1-d | 912 | 99.56 | 100 | 303 | 99.67 | 100 | |
| GCF_000149085 | LactinV 09V1-c | 912 | 99.56 | 100 | 303 | 99.34 | 100 | |
| GCF_000149105 | LactinV 03V1-b | 582 | 99.83 | 100 | 193 | 99.48 | 100 | |
| GCF_000149125 | LactinV 01V1-a | 780 | 99.49 | 100 | 259 | 99.15 | 90 | |
| GCF_000149145 | SPIN 2503V10-D | 912 | 99.67 | 100 | 303 | 99.67 | 100 | |
| GCF_000160875 | DSM 13335 | 912 | 99.56 | 100 | 303 | 99.67 | 100 | |
| GCF_000177755 | AB-1 | 912 | 99.56 | 100 | 303 | 99.67 | 100 | |
| GCF_000179935 | LEAF 2053A-b | 912 | 99.56 | 100 | 303 | 99.67 | 100 | |
| GCF_000179955 | LEAF 2052A-d | 912 | 99.67 | 100 | 303 | 99.67 | 100 | |
| GCF_000179975 | LEAF 2062A-h1 | 912 | 99.34 | 100 | 303 | 98.68 | 100 | |
| GCF_000179995 | LEAF 3008A-a | 912 | 99.67 | 100 | 303 | 99.67 | 100 | |
| GCF_000185405 | ATCC 55195 | 912 | 99.56 | 100 | 303 | 99.67 | 100 | |
| GCF_000191685 | UPII 143-D | 912 | 99.67 | 100 | 303 | 99.67 | 100 | |
| GCF_000191705 | UPII 60-B | 912 | 99.67 | 100 | 303 | 99.67 | 100 | |
| GCF_000204435 | SPIN 1401G | 912 | 99.56 | 100 | 303 | 99.67 | 100 | |
| GCF_000227195 | 7_1_47FAA | 912 | 99.67 | 100 | 303 | 99.34 | 100 | |
| GCF_001435015 | DSM 13335 | 912 | 99.56 | 100 | 303 | 99.67 | 100 | |
| GCF_002871595 | UMB0033 | 912 | 99.45 | 100 | 303 | 99.34 | 100 | |
| GCF_002884695 | UMB1051 | 912 | 99.45 | 100 | 303 | 99.67 | 100 | |
| GCF_002884705 | UMB0030 | 912 | 99.45 | 100 | 303 | 99.01 | 100 | |
| GCF_002892385 | KA00186 | 912 | 99.67 | 100 | 303 | 99.67 | 100 | |
| GCF_009556455 | LI335 | 912 | 99.56 | 100 | 303 | 99.67 | 100 | |
| GCF_902374445 | EMG | 912 | 99.67 | 100 | 303 | 99.34 | 100 | |

| **Accession** | **strain** | **hsdR** | | | | | |  |
| --- | --- | --- | --- | --- | --- | --- | --- | --- |
|  |  | **nucleotide length** | **Identities** | **Query Coverage** | **amino acids length** | **Identities** | **Query Coverage** | |
| GCF_000149065 | LactinV 11V1-d | 996 | 98.79 | 83 | 331 | 100 | 83 | |
| GCF_000149085 | LactinV 09V1-c | 1170 | 99.57 | 100 | 389 | 99.49 | 100 | |
| GCF_000149105 | LactinV 03V1-b | 966 + 213 | 98.55 | 86 | 321 + 70 | 100 | 86 | |
| GCF_000149125 | LactinV 01V1-a | 1116 | 99.52 | 74 | 371 | 100 | 74 | |
| GCF_000149145 | SPIN 2503V10-D | 3243 | 98.29 | 36 | 1080 | 99.23 | 36 | |
| GCF_000160875 | DSM 13335 | 1335 | 98.55 | 88 | 444 | 98.97 | 88 | |
| GCF_000177755 | AB-1 | 1335 | 98.55 | 88 | 444 | 98.97 | 88 | |
| GCF_000179935 | LEAF 2053A-b | 1170 | 98.46 | 100 | 389 | 97.94 | 100 | |
| GCF_000179955 | LEAF 2052A-d | 1731 | 97.35 | 68 | 576 | 97.17 | 68 | |
| GCF_000179975 | LEAF 2062A-h1 | 1170 | 99.83 | 100 | 389 | 100 | 100 | |
| GCF_000179995 | LEAF 3008A-a | 1170 | 99.83 | 100 | 389 | 100 | 100 | |
| GCF_000185405 | ATCC 55195 | 1731 | 97.35 | 68 | 576 | 97.17 | 68 | |
| GCF_000191685 | UPII 143-D | 1170 | 99.83 | 100 | 389 | 100 | 100 | |
| GCF_000191705 | UPII 60-B | 1170 | 98.8 | 100 | 389 | 99.23 | 100 | |
| GCF_000204435 | SPIN 1401G | 1731 | 98.38 | 68 | 576 | 99.23 | 68 | |
| GCF_000227195 | 7_1_47FAA | 996 | 99.52 | 83 | 331 | 100 | 83 | |
| GCF_001435015 | DSM 13335 | 1335 | 98.55 | 88 | 444 | 98.97 | 88 | |
| GCF_002871595 | UMB0033 | 831 | 97.95 | 99 | 276 | 98.55 | 100 | |
| GCF_002884695 | UMB1051 | 996 | 98.55 | 83 | 331 | 100 | 83 | |
| GCF_002884705 | UMB0030 | 1170 | 98.21 | 100 | 389 | 97.94 | 100 | |
| GCF_002892385 | KA00186 | 915 | 99.56 | 100 | 304 | 99.34 | 100 | |
| GCF_009556455 | LI335 | 1335 | 98.55 | 88 | 444 | 98.97 | 88 | |
| GCF_902374445 | EMG | 996 | 99.52 | 83 | 331 | 100 | 83 | |
